# Microbial production and characterization of poly-3-hydroxybutyrate by *Neptunomonas antarctica*

**DOI:** 10.7717/peerj.2291

**Published:** 2016-08-02

**Authors:** Xiao-Jie Liu, Jie Zhang, Peng-Hui Hong, Zheng-Jun Li

**Affiliations:** Beijing Key Laboratory of Bioprocess, College of Life Science and Technology, Beijing University of Chemical Technology, Beijing, China

**Keywords:** Halophilic, Neptunomonas antarctica, Poly-3-hydroxybutyrate, Seawater, Marine bacteria

## Abstract

Considering the industrial interest of biodegradable polymer poly-3-hydroxybutyrate (PHB), the marine bacteria *Neptunomonas antarctica* was studied for its ability to accumulate PHB. The extracted polymer was confirmed to be PHB by nuclear magnetic resonance analysis. In shake flask cultures using natural seawater as medium components, PHB was produced up to 2.12 g/L with a yield of 0.18 g PHB/g fructose. In the presence of artificial seawater, the PHB titer and yield reached 2.13 g/L and 0.13 g PHB/g fructose, respectively. The accumulated polymer gradually decreased when fructose was exhausted, indicating that intracellular PHB was degraded by *N. antarctica*. The weight-average and number-average molecular weights of PHB produced within natural seawater were 2.4 × 10^5^ g/mol and 1.7 × 10^5^ g/mol, respectively. Our results highlight the potential of *N. antarctica* for PHB production with seawater as a nutrient source.

## Introduction

Poly-3-hydroxybutyrate (PHB) is a microbially produced biodegradable polymer and regarded as an alternative to conventional petrochemical plastics (Chen, 2009). The species of *Bacillus*, *Alcaligenes* and *Pseudomonas* are most common natural PHB producers, and the intracellular polymer was reported to be accumulated up to 90 wt% of cell dry weight under nutrients limited conditions (Prabisha et al., 2015). The application of eco-friendly PHB as commodity plastics could alleviate the waste disposal problems and carbon dioxide emissions. Nevertheless, the present price of PHB is not feasible to replace traditional petro-based plastics, and the high production cost imposes great restrictions on the commercialization of PHB material (RamKumar Pandian et al., 2010).

The inexpensive substrates such as crude glycerol and agricultural wastes have been explored for PHB production to reduce the feedstock cost (Wei et al., 2015). On the other hand, the open unsterile and continuous fermentation process was also developed to lower PHB production cost, which employed halophiles as producers given that high salts concentration prevents the growth of nonhalophilic microorganisms (Fu et al., 2014; Li et al., 2014; Tan et al., 2011). Previous studies showed that *Halomonas boliviensis* and *Halomonas* TD01 were two promising moderate halophilic hosts for efficient PHB production. In fed-batch cultivation process, PHB could accumulate up to 80 wt% of cell dry weight, and cell dry weight reach 44 g/L and 80 g/L, respectively (Quillaguaman et al., 2008; Quillaguaman et al., 2010; Tan et al., 2011).

The marine environments provide an exciting resource for the discovery of new classes of PHB producers. Most marine bacteria are halophilic microorganisms and require NaCl for their growth. Several strains of *Vibrio* spp. isolated from marine environments were studied for their potential of PHB production, and the highest polymer titer was 4.2 g/L, which was achieved in a batch cultivation using 3 l bioreactor. However, there are very few reports on marine PHB producers other than *Vibrio* spp (Arun et al., 2009; Chien et al., 2007; Sun et al., 1994; Weiner, 1997). Therefore, we decided to attempt to identify new PHB-producing marine bacteria. In this study, we described a novel cryophilic and halophilic PHB producing strain, *Neptunomonas antarctica*, which was isolated from Antarctica marine sediment (Zhang et al., 2010). Thereafter, the polymer produced by *N. antarctica* was characterized by nuclear magnetic resonance (NMR) analysis and molecular weight assay.

## Materials and Methods

### Strain and culture conditions

*N. concharum* JCM 17730, *N. qingdaonensis* CGMCC 1.10971 and *N. antarctica* CCTCC AB 209086 were purchased from Japan Collection of Microorganisms, China General Microbiological Culture Collection Center and China Center for Type Culture Collection, respectively. The three species of marine bacteria were cultivated at optimal temperatures in TYS broth medium. TYS broth medium contained (g/L) Bacto tryptone 5, yeast extract 1, dissolved with artificial seawater (2.75% NaCl, 0.07% KCl, 0.54% MgCl_2_⋅6H_2_O, 0.68% MgSO_4_⋅7H_2_O, 0.14% CaCl_2_⋅2H_2_O, 0.02% NaHCO_3_ in distilled water). For PHB producing experiments, strains were firstly incubated in TYS medium and shaken at 150 rpm to prepare seed cultures. Subsequently, 5% (*v*∕*v*) seed culture was inoculated into 500 ml shake flasks containing 100 ml TYS or TYSN medium supplemented with fructose and shaken at 150 rpm. TYSN medium contained (g/L) Bacto tryptone 5, yeast extract 1, dissolved with natural seawater (collected from Bohai Bay, Tianjin, China).

### Measurements of cell growth and PHB production

At three-day intervals, 1 ml of culture was sampled from the Erlenmeyer flasks for cell optical density (OD) analysis. For cell dry weight (CDW) measurements, strain cultures were harvested by centrifugation at 10,000 g, washed with artificial seawater and distilled water respectively, and then lyophilized to constant weight. To measure the intracellular polymer content, lyophilized cells were subjected to methanolysis at 100 °C for 4 h in the presence of 3% (*v*∕*v*) H_2_SO_4_ in a screw-capped tube and then assayed with a gas chromatograph equipped with the DB-5 capillary column (Agilent) and a hydrogen flame ionization detector. PHB purchased from Sigma-Aldrich was used as standards. For fructose measurements, the supernatant of cell cultures was filtered through a 0.2-µm syringe filter and then analyzed by high-performance liquid chromatography equipped with an ion exchange column (Aminex HPX-87H) and a refractive index detector. The column was maintained at 50 °C and 5 mM of sulfuric acid was used as the mobile phase at the flow rate of 0.6 ml/min.

### Extraction and characterization of PHB polymers

PHB polymers were extracted from the lyophilized bacteria cells with chloroform in screw-capped tubes at 100 °C for 4 h. An equal volume of distilled water was added into the tubes and vortexed, subsequently, the chloroform phase was collected by centrifugation at 10,000 g, and then precipitated in an excess of 10 volumes of ice-cold ethanol. The PHB sediment was collected *via* centrifugation and vacuum drying for 24 h. Dried PHB samples were dissolved with deuterated chloroform (CDCl_3_) to study the structural elucidation. The ^1^H and ^13^C NMR spectrum of PHB polymer were recorded on a JEOL JNM-ECA500 spectrometer. The molecular weights of PHB polymers were detected by gel permeation chromatography equipped with Shodex K-804 column (Waters). Chloroform was used as the eluent at a flow rate of l ml/min, and polymer sample concentrations of 5 mg/ml were applied. The polystyrene standards purchased from Sigma-Aldrich were used as standards for calibration.

## Results and Discussion

### Selection of *Neptunomonas* for polymer accumulation

The genus *Neptunomonas* comprises a group of Gram-negative, rod-shaped, non-fermentative and aerobic marine bacteria (Hedlund et al., 1999). A variety of *Neptunomonas* members were discovered and characterized in the past few years, and most of them were isolated from sea and ocean environments (Yang et al., 2014). Three species of * Neptunomonas*, including *N. concharum* (Lee et al., 2012), *N. qingdaonensis* (Liu et al., 2013) and *N. antarctica* (Zhang et al., 2010) were cultivated with TYS medium supplemented with glucose or fructose as carbon source to study their polymer producing ability. The lyophilized bacteria cells were subjected to PHB extraction process. *N. antarctica* was speculated to be capable of polymer accumulation, while *N. concharum* and *N. qingdaonensis* could not produce any polymer.

### The identification of polymer produced by *N. antarctica*

**Figure 1:**
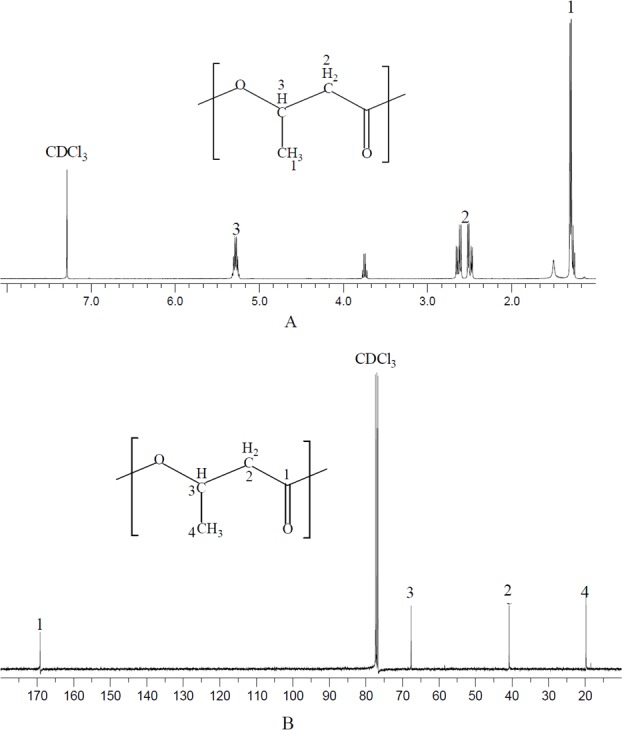
NMR spectrum of polymer produced by *N. antarctica*. Intracellular polymer was extracted from the 9-day shake flask culture of *N. antarctica*. (A) ^1^H NMR spectrum; (B) ^13^C NMR spectrum.

NMR spectrum provides detailed structure information for polymer characterization. The polymer produced by *N*. *antarctica* was extracted and dissolved with CDCl_3_ for NMR analysis (Fig. 1). As shown in the ^13^C NMR spectrum, four types of carbon atoms were observed, which were consistent with the structure of PHB: carbonyl (C=O), methane (CH), methylene (CH_2_), and methyl (CH_3_) groups. Moreover, the chemical shift signals of the polymer product were the same as PHB reported in previous studies (Doi et al., 1986). In terms of ^1^H NMR spectrum, three groups of signals characteristic of PHB homopolymer were observed. The doublet at 1.2–1.3 ppm was attributed to the methyl group; the doublet of the quadruplet at 2.4–2.7 ppm to the methylene group and the singlet at 5.2–5.3 ppm to the methylene group (Doi et al., 1986). The above analysis clearly confirmed that the polymer extracted from *N. antarctica* was PHB homopolymer.

### PHB production profile of *N. antarctica* in shake flasks

**Figure 2:**
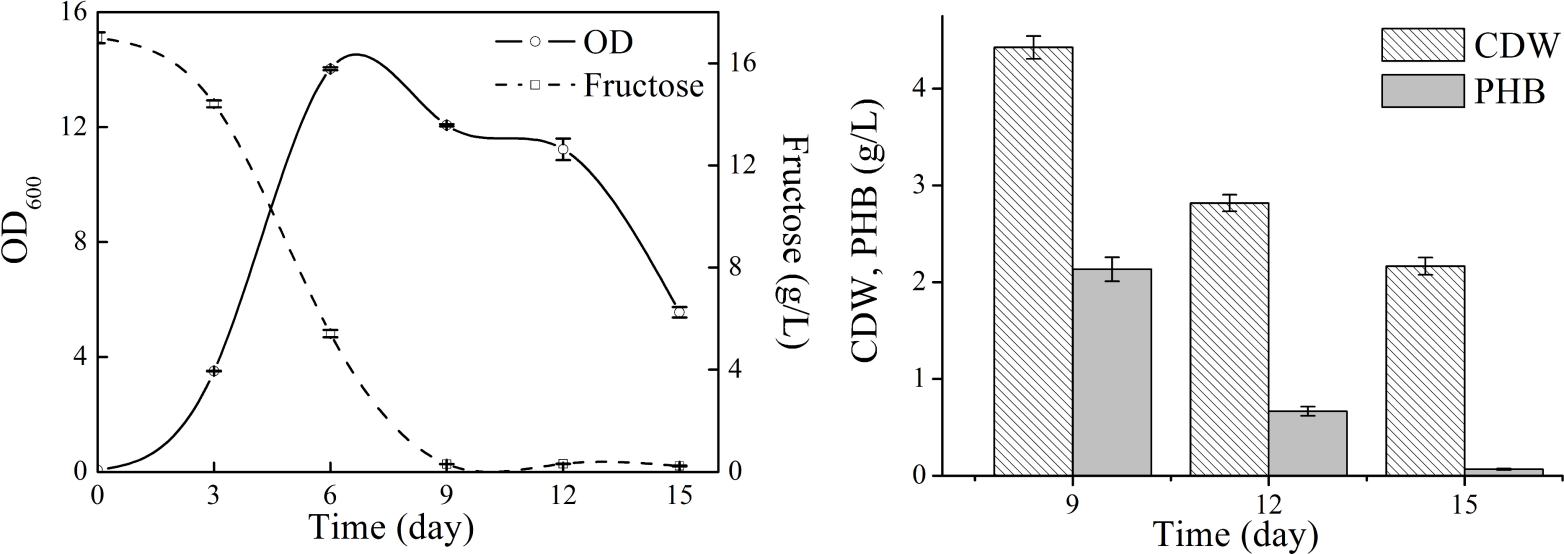
Cell growth, fructose consumption and PHB accumulation profile of *N. antarctica* cultivated in TYS medium. Bacteria were cultivated in 500 ml Erlenmeyer flasks containing 100 ml medium at 15 °C and 150 rpm. Optical density (OD) was measured at 600 nm. Data were expressed as average value and standard deviation of three parallel samples. Initial fructose concentration was 17.0 g/L.

Detailed cell growth and PHB production profile of *N. antarctica* was studied with TYS medium (Fig. 2). The strain exhibited rapid growth at the first 6 days and then slowed down to stationary and decline phase. The maximum OD was approximately 14, and the highest PHB titer was 2.13 g/L with a yield of 0.13 g PHB/g fructose. The PHB content gradually decreased when the strain consumed all carbon sources and only 3 wt% PHB was left after 15-day cultivation, which indicated that the accumulated PHB was subjected to degradation in the late stationary phase.

**Figure 3:**
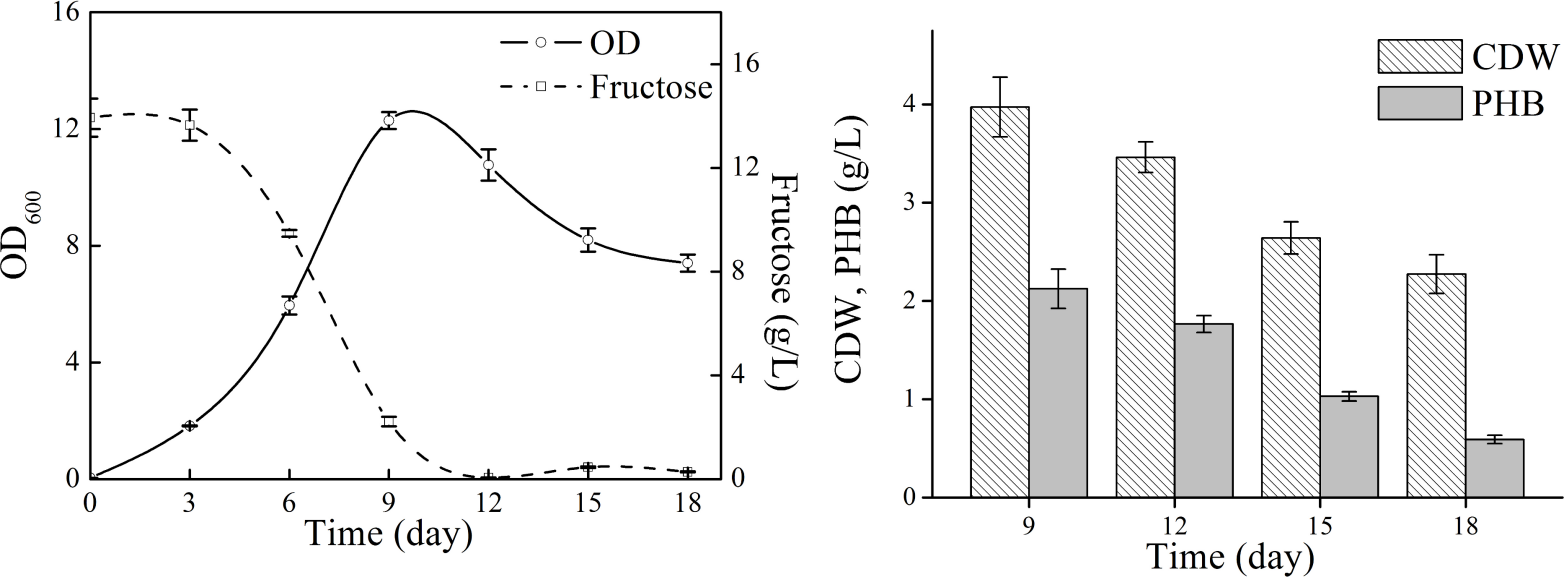
Cell growth, fructose consumption and PHB accumulation profile of *N. antarctica* cultivated in TYSN medium. Bacteria were cultivated in 500 ml Erlenmeyer flasks containing 100 ml medium at 15 °C and 150 rpm. Optical density (OD) was measured at 600 nm. Data were expressed as average value and standard deviation of three parallel samples. Initial fructose concentration was 14.0 g/L.

Next, natural seawater was applied to replace the mineral salts to perform shake flask experiments (Fig. 3). The cell growth in TYSN medium was slightly lower than that obtained in TYS medium, and OD reached its highest point at around day 9 and fructose was used up after 12-day cultivation. The maximum PHB titer was 2.12 g/L, which was close to the data obtained in TYS medium. In terms of PHB production yield, natural seawater (0.18 g PHB/g fructose) was superior to artificial seawater. Moreover, the final PHB content was 26 wt%, indicating that the produced polymer was much more stable when natural seawater was employed as medium components. The PHB degradation rate was 0.17 g/L/day, much lower than that of 0.34 g/L/day obtained in TYS medium. Previously, raw seawater was used as a cost-free source of mineral salts for PHB production by *Bacillus megaterium* (RamKumar Pandian et al., 2010). Here, we report for the use of natural seawater as a nutrient source by marine bacteria *N. antarctica*.

We next attempted to investigate the possibility of the unsterile process for PHB production, in which the shake flasks and initial media were not sterilized (Fig. S1). The highest PHB production titer was only 0.1 g/L, indicating that the cultures were contaminated. Previously, the moderate halophile *Halomonas* TD01 was proved to be suitable for the open and continuous PHB fermentation process, and the NaCl concentration in the medium was 60 g/L (Tan et al., 2011). However, the optimal NaCl concentration for *N. antarctica* is much lower than that for *Halomonas*. Moreover, *N. antarctica* is a cryophilic bacteria and has a lower growth rate, which is a disadvantage to compete with the contaminating bacteria. Therefore, the unsterile process might not be feasible for the cryophilic and halophilic marine bacteria *N. antarctica.*

### Molecular weight assay of PHB

The molecular weight assay of PHB produced by *N. antarctica* was performed (Table 1). The weight-average molecular weight (*M*_*w*_) and number-average molecular weight (*M*_*n*_) of PHB cultivated with TYS medium were 1.9 × 10^5^ g/mol and 1.4 × 10^5^ g/mol, respectively. With natural seawater as nutrients, PHB molecular weight was slightly increased. *M*_*w*_ and *M*_*n*_ reached up to 2.4 × 10^5^ g/mol and 1.7 × 10^5^ g/mol, respectively. The polydispersity index (*M*_*w*_/*M*_*n*_) for the two samples were 1.3 and 1.4, respectively, indicating narrow molecular weight distributions. It has been proposed that polyhydroxyalkanoate synthase activity and host bacteria are two major factors controlling the polymer molecular weight (Sim et al., 1997). The molecular weights of PHB obtained from *N. antarctica* were lower than polymers produced by another halophilic bacterium termed *Halomonas* or the most common PHB producing strain *Ralstonia eutropha* (Tan et al., 2011; Tanadchangsaeng & Yu, 2012).

**Table 1:** Molecular weights of PHB synthesized by *N*. *antarctica* and other producers.

Strain	Medium	Molecular weight		
		*M*_*w*_(×10^5^ g/mol)	*M*_*n*_(×10^5^ g/mol)	*M*_*w*_/*M*_*n*_
*N*. *antarctica*	TYS	1.9	1.4	1.3
*N*. *antarctica*	TYSN	2.4	1.7	1.4
*Halomonas*^a^	–	6	–	–
*R. eutropha*^b^	Mineral solution and glycerol	5.5	2.8	2.0

**Notes.**

*M*_*w*_ weight-average molecular weight *M*_*n*_ number-average molecular weight

a Tan et al. (2011).

b Tanadchangsaeng & Yu (2012).

## Conclusions

The study on PHB production using halophilic bacteria has attracted growing interest because of the advantage of unsterile and continuous fermentation process. However, few reports have been published to explore the potential of PHB production using marine bacteria and seawater as nutrient sources. In this study, we reported the potential of *N. antarctica* for PHB production with seawater as nutrient sources. In shake flask cultures, PHB was produced up to a maximum titer of 2.12 g/L with a yield of 0.18 g PHB/g fructose. The structure of produced polymer was confirmed by NMR analysis. Upcoming research on cultivation optimization should achieve the low cost PHB production process.

##  Supplemental Information

10.7717/peerj.2291/supp-1 Figure S1 Cell growth and PHB accumulation profile of *N. antarctica* cultivated in unsterile condition Bacteria were cultivated in 500 ml erlenmeyer flasks containing 100 ml medium at 15 °C and 150 rpm. The shake flasks and initial media were not sterilized.

10.7717/peerj.2291/supp-2 Data S1 Raw data for Figs

## Additional Information and Declarations

### Data Availability

The following information was supplied regarding data availability: The raw data has been supplied as Data S1.
