# Supplementary figures and images for: Microbial production and characterization of poly-3-hydroxybutyrate by Neptunomonas antarctica
Source: PeerJ. 2016 Aug 2;4:e2291. doi: 10.7717/peerj.2291 (PMC4975036; doi:10.7717/peerj.2291)

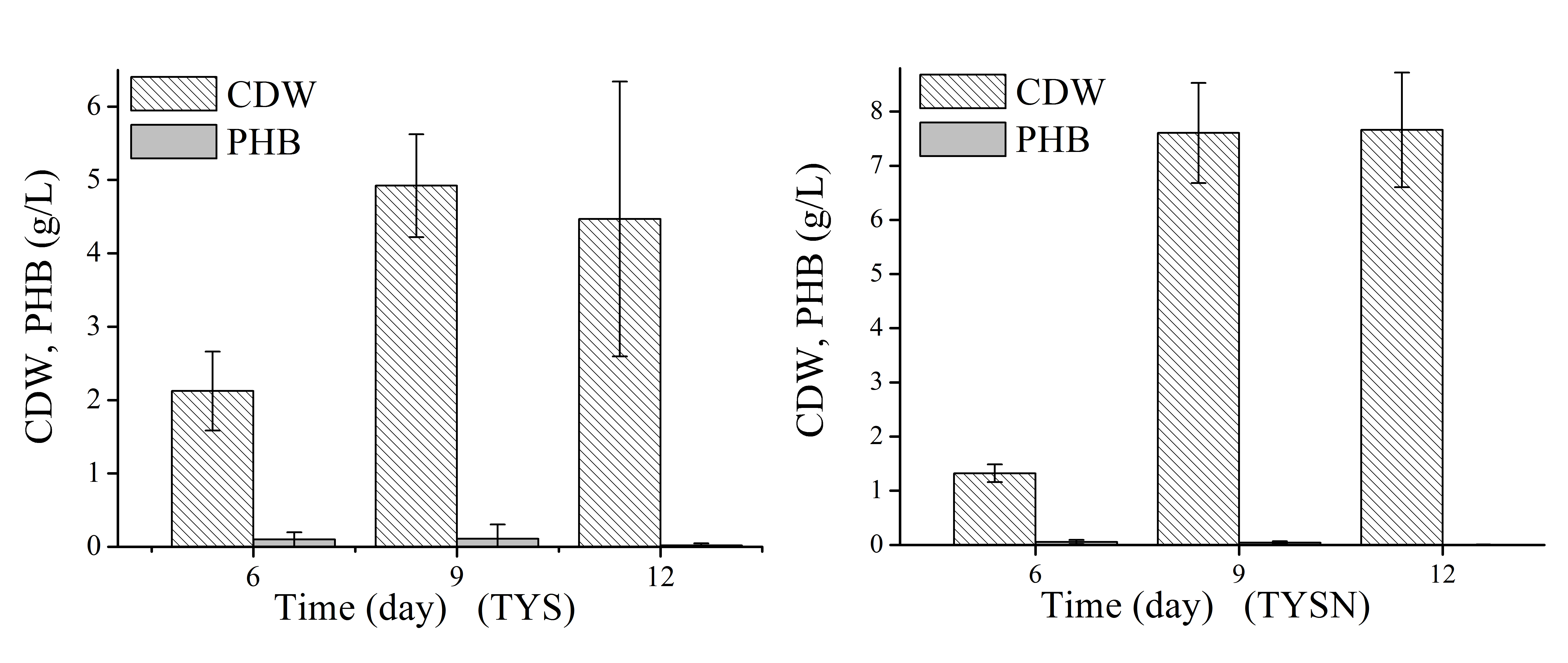

Supplement: Figure S1 — Bacteria were cultivated in 500 ml erlenmeyer flasks containing 100 ml medium at 15 °C and 150 rpm. The shake flasks and initial media were not sterilized. [file peerj-04-2291-s001.png]
