# Supplementary material for: Microbial production and characterization of poly-3-hydroxybutyrate by Neptunomonas antarctica
Source: PeerJ. 2016 Aug 2;4:e2291. doi: 10.7717/peerj.2291 (PMC4975036; doi:10.7717/peerj.2291)
Supplement: Data S1 [file peerj-04-2291-s002.docx]

Data for Fig. 2

| day | OD | SD | CDW  (g/L) | SD | PHB  (g/L) | SD | Fructose  (g/L) | SD | Yield of PHB |
| --- | --- | --- | --- | --- | --- | --- | --- | --- | --- |
| 0 | 0.07 | 0.00 |  |  |  |  | 17.00 | 0.21 |  |
| 3 | 3.51 | 0.01 |  |  |  |  | 14.42 | 0.13 |  |
| 6 | 14.03 | 0.05 |  |  |  |  | 5.42 | 0.14 |  |
| 9 | 12.06 | 0.04 | 4.43 | 0.12 | 2.13 | 0.12 | 0.31 | 0.00 | 0.13 |
| 12 | 11.23 | 0.37 | 2.82 | 0.09 | 0.67 | 0.05 | 0.31 | 0.00 | 0.04 |
| 15 | 5.56 | 0.19 | 2.16 | 0.09 | 0.07 | 0.01 | 0.23 | 0.02 | 0.00 |

Data for Fig. 3

| day | OD | SD | CDW  (g/L) | SD | PHB  (g/L) | SD | Fructose  (g/L) | SD | Yield of PHB |
| --- | --- | --- | --- | --- | --- | --- | --- | --- | --- |
| 0 | 0.05 | 0.00 |  |  |  |  | 13.98 | 0.74 |  |
| 3 | 1.83 | 0.02 |  |  |  |  | 13.65 | 0.60 |  |
| 6 | 5.96 | 0.31 |  |  |  |  | 9.48 | 0.13 |  |
| 9 | 12.29 | 0.29 | 3.97 | 0.30 | 2.12 | 0.20 | 2.22 | 0.18 | 0.18 |
| 12 | 10.77 | 0.54 | 3.46 | 0.16 | 1.77 | 0.09 | 0.06 | 0.00 | 0.12 |
| 15 | 8.20 | 0.40 | 2.64 | 0.16 | 1.03 | 0.05 | 0.46 | 0.03 | 0.06 |
| 18 | 7.41 | 0.29 | 2.27 | 0.20 | 0.59 | 0.04 | 0.28 | 0.02 | 0.04 |

Data for Fig. S1

| TYS | | | | |
| --- | --- | --- | --- | --- |
| day | CDW(g/L) | SD | PHB(g/L) | SD |
| 6 | 2.12 | 0.54 | 0.10 | 0.10 |
| 9 | 4.92 | 0.70 | 0.11 | 0.19 |
| 12 | 4.47 | 1.87 | 0.02 | 0.03 |

| TYSN | | | | |
| --- | --- | --- | --- | --- |
| day | CDW(g/L) | SD | PHB(g/L) | SD |
| 6 | 1.32 | 0.16 | 0.05 | 0.04 |
| 9 | 7.60 | 0.92 | 0.04 | 0.03 |
| 12 | 7.66 | 1.06 | 0.00 | 0.00 |
